# The barriers of home environments for obesity prevention in Indonesian adolescents

**DOI:** 10.1186/s12889-022-14669-6

**Published:** 2022-12-14

**Authors:** Fransisca Handy Agung, Rini Sekartini, Nani Cahyani Sudarsono, Aryono Hendarto, Meita Dhamayanti, Retno Asti Werdhani, Susan M. Sawyer

**Affiliations:** 1 Present address: Doctoral Program Faculty of Medicine Universitas Indonesia, Jl. Salemba Raya No.6 Jakarta Pusat, Jakarta, 10430 Indonesia; 2 Faculty of Medicine, Universitas Pelita Harapan, Jl. Jend. Sudirman No.20, Bencongan, Kelapa Dua, Tangerang, Banten 15810 Indonesia; 3 Department of Child Health, Faculty of Medicine Universitas Indonesia, Jl. Salemba Raya No.6 Jakarta Pusat, Jakarta, 10430 Indonesia; 4 Department of Community Medicine, Faculty of Medicine Universitas Indonesia, Jl. Pegangsaan Timur no 16 Jakarta Pusat, Jakarta, 10310 Indonesia; 5 Department of Child Health, Faculty of Medicine Universitas Padjajaran, Jl. Professor Eyckman No.38, Pasteur, Bandung, West Java Indonesia; 6 Centre for Adolescent Health, Royal Children’s Hospital, Murdoch Children’s Research, Institute, Department of Paediatrics, University of Melbourne, 50 Flemington Rd, Parkville, VIC 3052 Australia

**Keywords:** Adolescent, Eating behaviours, Healthy lifestyles, Obesity, Parenting, Physical activity, Prevention

## Abstract

**Background:**

Obesity and its related cardiovascular-metabolic diseases are growing public health concerns. Despite global attention to obesity, its prevalence is steeply increasing in developing countries, especially in children and adolescents. Eating behaviours and physical activity are modifiable risk factors for obesity that can variably be shaped by families. Eating behaviours and physical activity are especially important during adolescence, given its significance as a foundational period for developing healthy lifestyles. This qualitative study aimed to explore barriers and opportunities around creating healthy lifestyles among adolescents in Indonesia, focussing on family environments from diverse socio-demographic backgrounds.

**Method:**

In-depth interviews using a semi-structured guide were undertaken with consecutively recruited 10–18-year-old adolescents with overweight or obesity, and their parents, from three different sites: urban (Jakarta, the capital city of Indonesia), peri-urban (West Java Province) and rural (Banten Province). Thematic analysis was used to identify patterns of meaning.

**Results:**

Nineteen dyads were interviewed. Thematic analysis revealed four themes: limited knowledge of healthy lifestyles; healthy lifestyles not a concern of daily life; limited parenting skills, including inequity around gender roles; and aspects of availability and accessibility. These interconnected barriers influenced lifestyle practices at home within the context of daily preferences and decisions around food and activities. Gender role inequity and healthy food accessibility were more prominent in rural families than in those from urban or peri-urban settings.

**Conclusions:**

Healthy lifestyles in adolescence may be supported by strategies to enhance parenting skills, build individual motivation, and support the development of more enabling environments.

## Introduction

Over the past four decades, there has been a ten fold increase in the global prevalence of obesity in adolescents [1]. Many low and middle-income countries have experienced an even more rapid increase [2]. In Indonesia, for example, a 2018 national health survey revealed 19.8% of adolescents were overweight and obesity, a ten fold increase in the past decade [3, 4]. The COVID-19 pandemic is expected to amplify this trend due to many life restrictions [5].

Overnutrition impacts adolescents’ health and wellbeing. Adolescents with overweight or obesity have a five to eight times greater risk of developing cardiovascular-metabolic problems in later adulthood [6, 7]. In the past deacde, cardiovascular-metabolic diseases such as stroke, heart disease and diabetes have caused the greatest morbidity and mortality in Indonesia than other disease groups, and contributing to rising health costs and reduced productivity [8].

Healthy diet and physical activity are central to preventing overweight and obesity [9]. A recent systematic review of fifteen studies of adolescents in Indonesia highlighted the poor quality of their diet [10]. All of the reviewed studies revealed inadequate fruit and vegetable consumption, high consumption of sugary beverages, and frequent skipping of breakfast and snacking among 10–17-year-olds [10]. Furthermore, the 2018 national survey in Indonesia showed that in terms of physical activity, the proportion of the population over 10 years of age within the ‘less active’ category was 33.7% [4]. Higher than recorded in the same survey from only 5 years earlier [3], physical inactivity is expected to have increased even further during the COVID-19 pandemic [11].

Parent capabilities to provide enabling environments for children to develop a healthy lifestyle will reflect a variety of barriers and facilitators in homes as well as in communities. While we know ‘what’ family factors affect adolescent lifestyles, it is not entirely clear ‘how’ and ‘why’ these factors are, or are not, able to support adolescents in adopting healthy lifestyle practices, especially in Asia [12]. Certainly, there is scarce evidence about how Indonesian parents understand their role and what factors influence them around shaping healthy behaviors in their children. For this reason, we aimed to explore the barriers and opportunities around healthy eating behaviors and physical activity among Indonesian adolescents and their parents across various sociodemographic contexts. We focused on the home environment, as we wished to explore how parents understand their roles and responsibilities around promoting healthy lifestyles in their children, and the practices they engage in within their daily lives. The wider objective of this study was to use these findings to inform the development of a training module for primary healthcare professionals.

## Method

This is a qualitative study using semi-structured in-dept interviews to adolescents and their parents.

### Development of the interview guide

A semi-structured interview guide was developed in the Indonesian language (Bahasa) by the lead researcher, in consultation with the group. This was refined after pilot interviews with two adolescents (a boy and a girl) and two parents (a mother and a father). The interview questions focused on three key areas: knowledge of healthy eating, physical activity, sedentary activity (screen time) and obesity; daily life-style practices at home; and the parent-adolescent relationship, especially around the establishment of healthy lifestyles at home (see Table 1).

**Table 1:** Interview guide

Area	Examples of questions
1. Knowledge on obesity and healthy lifestyle	- What do you know about obesity and its impacts?
	- What is healthy eating? What is healthy snacking?
	- What are the recommendations for physical activity and screen time for adolescents?
2. Daily practices at home and the modifiable factors around these	- What are your eating habits at home?
	- What are your daily activities?
	- How many hours screen time do you get each day?
	- What are the factors that influence your eating habits / activities / screen time habits?
3. Parent-adolescent relationship around healthy lifestyle establishment	- Is there any discussion or talk on healthy lifestyles at home?
	- What are the house rules about eating / physical activity / screen time?
	- How are meals organised at home?
	- How are daily household chores organised at home?
	- How do you understand and apply your role as a parent?

### Settings and participants

Participants were adolescents and one of their parents who were enrolled between December 2019–February 2020 from three different socio-demographic backgrounds: an urban setting (Jakarta, the capital city of Indonesia), a peri-urban setting (Bogor, a district near Jakarta in West Java Province), and a rural setting (Pandeglang, a rural district in Banten Province). The inclusion criteria were that adolescents were aged 10–18 years old, had a BMI percentile of 85 or above (WHO growth chart 2006), did not have any developmental concerns and lived with their parents.

### Recruitment

For each location, a local research assistant experienced in community-based research was recruited to identify potentially eligible participants. Those from the peri-urban and rural settings were identified with communication with the local leader. In the urban setting, participants were also recruited using social media (Facebook, Whatsapp). The research assistant provided a thorough explanation of the study to potential participants by phone or face-to-face. If the potential participants and one of his/her parents expressed interest in participating in the study, an interview was arranged, prior to which a brief explanation was repeated in person by the interviewer at the scheduled interview time before participants were invited to provide signed, informed consent/assent. We hoped to recruit a gender balance of boys and girls and to recruit at least one father from each location (expecting that the majority of participating parents would be mothers). A convenience sampling method was used, with recruitment continuing until no new codes were found in the data transcripts.

### Data collection

Three trained interviewers with experience communicating with adolescents used a semi-structured guide to undertake the interviews at the study participants’ homes. One interviewer was used for each of the three population groups. In the peri-urban and rural populations, each interview also included the local research assistant to help translate local languages and local customs. Each interview started with a brief explanation of the study, following which written consent was obtained from the parent and assent from the adolescent. Each adolescent and their parents then separately completed a demographic questionnaire. After a statement that suggested they may be more comfortable being interviewed alone, they were offered the opportunity to be interviewed alone or together. Each interview took approximately 45 to 60 minutes. All interviews were audio-recorded and transcribed for analysis in Bahasa, and also translated into English.

### Data analysis

The transcribed interviews were analysed by the lead researcher and two independent analysts with different backgrounds: a health communication expert with expertise in adolescent health advocacy, and a general practitioner with extensive counseling experience around nutrition and family wellbeing in rural communities. A line-by-line open coding process was used until thematic categories emerged. Each analyst generated an initial coding scheme that included a range of observable categories, following which the themes were then discussed among all the analysts. Once they were finalized, transcripts were imported into NVivo (Version 12.6.0, QSR International - UK) for electronic coding. Using the thematic coding frame, a chart of the data was created with each participant represented across all themes. This enabled comparison to be made within and across the interviews, allowed identification of deviant cases, and highlighted the views that participants held towards a particular issue [13].

## Results

Nineteen adolescents and their parents participated in the study, with an even balance across the urban (seven dyads), peri-urban (six dyads) and rural sites (six dyads). There were eight and eleven adolescents with overweight and obesity respectively. The group was evenly balanced between younger adolescents (nine adolescents were aged between 10 and 14 years) and older adolescents (ten were aged 15–19 years old). There were similar numbers of male and female adolescents. On the parent side, there were significantly more mothers (fourteen) than fathers (five). Parents from the rural area had all graduated from secondary school, those from the peri-urban area were a mix of secondary school and tertiary graduates, while those from the urban area all had tertiary qualifications. About half of the adolescents, mainly the younger ones, wished their parents to be present throughout the interview younger adolescents and parents from the rural area provided simpler responses in comparison to older adolescents, and parents from the urban setting were generally more expansive.

**Fig. 1:**
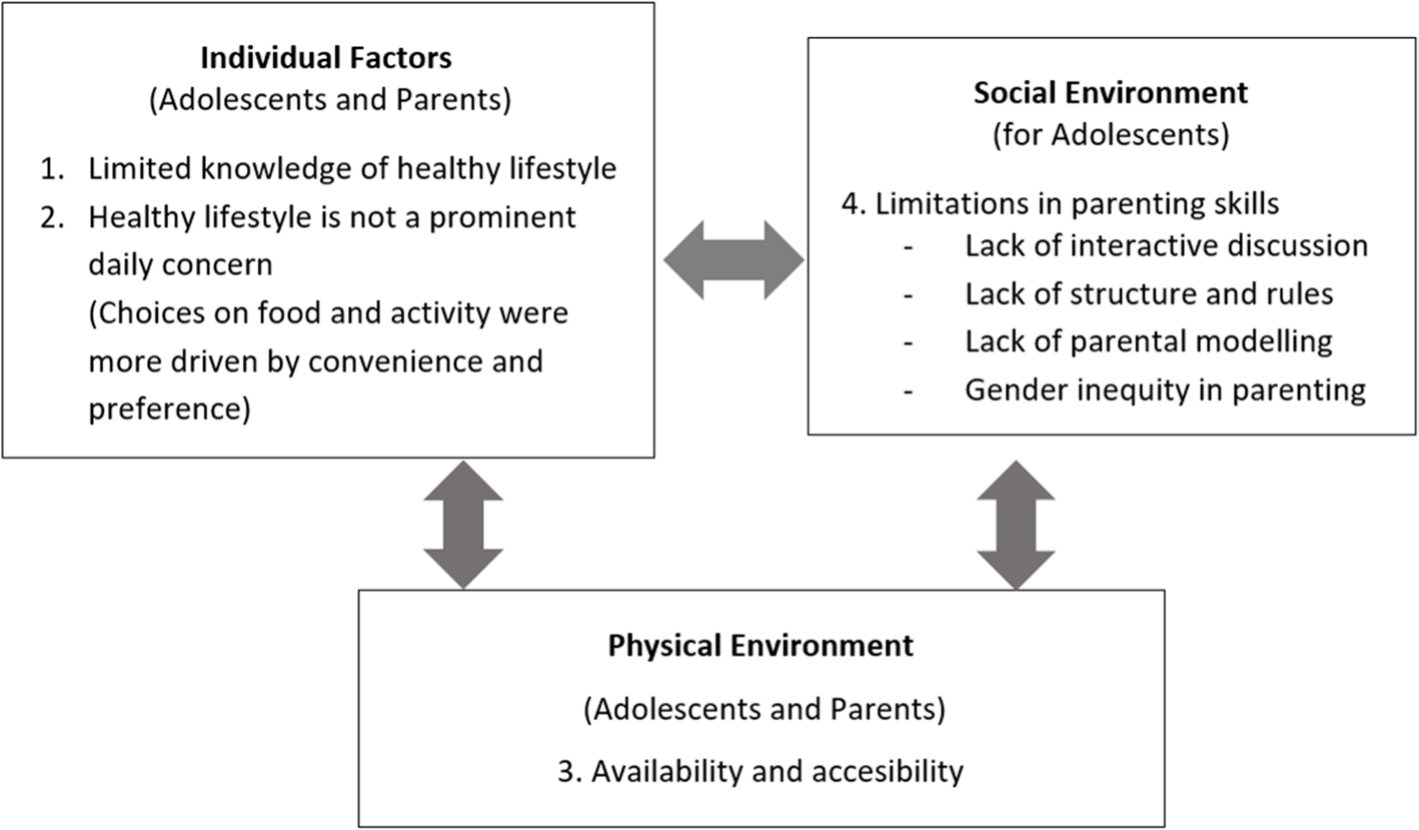
Thematic map depicting individual, social environment and physical environment factors associated with dietary and physical activity behaviours in adolescents

Four interdependent themes emerged from the analysis, as depicted in Fig. 1. Two themes (knowledge and personal concern) were considered to be individual factors for both adolescents and parents. The third theme (availability and accessibility) was considered to lie within the physical environment, and was also relevant for both adolescents and parents. The fourth theme (parenting skills) was considered to reflect the social environment experienced by adolescents.

### Theme 1. Limited knowledge and understanding

Participants from all three sites had some appreciation of the issues relating to healthy eating, with deeper, more explorative answers obtained from the urban population (see Table 2). Most study participants had some basic knowledge of healthy eating, such as the value of eating a range of types of food and the importance of regular mealtimes.

“Healthy eating means we eat regularly*”* (Mother, peri-urban)

*“*A healthy diet is a balanced diet, carbohydrates, protein, vitamins, minerals, and fiber” (Mother, urban)

**Table 2:** Representative quotes of theme 1 (knowledge of healthy eating, physical activity, screen time, overweight and obesity)

**Knowledge of healthy eating**	
Balanced nutrition is ‘healthy four perfect five’ (Girl, rural)	
I’ve never heard of a balanced diet (Father, rural)	
Well, as a saying ‘healthy four perfect five’, there have to be vegetables, carbohydrates, fruits, and milk.” (Boy, peri-urban)	
(Healthy eating is) we eat regularly and not too much (Mother, peri-urban)	
If we eat food that is oily or fatty, we can eat fruits and vegetables as neutralizers. (Question) What is a neutralizer? (Answer) It Something that is good for the body (Boy, peri-urban)	
Food that is balanced in nutrition, protein, carbohydrates, and vitamins. It does not mean there is no carbohydrate. Therefore, the food is balanced, not too much (Boy, urban)	
A healthy diet is no carbohydrates or fewer carbohydrates. A balanced diet includes healthy four perfect five.” (Mother, urban)	
(Question) What is food that contains fat? What is the source of fat? (Answer) Something fried (Question) Besides that, is there anything else (Answer) I do not know. (Boy, rural)	
Cola also contains a lot of sugar. Energy drinks and bottled drinks also contain a lot of sugar, especially for those who like snacks. Teh Sisri (a brand of bottled tea) or something like that also contains a lot of sugar. (Mother, peri-urban)	
(Question) Why fruits and vegetables are healthy food? (Answer) Because fruit and vegetables contain vitamins. Vitamins are important for our bodies.” (Girl, peri-urban)	
Fat comes from protein and also meat. Fish and chicken also contain fat. Moreover, there is also fat in plants. Healthy fat is said to be mostly in fish.” (Mother, urban)	
(Question) How much is should a teenager eat? (Answer) Well, when she/he feels full. (Father, peri-urban)	
**Knowledge of Physical Activity and Screen time**	
Physical activity is volleyball, swimming, jogging, and taking a walk and playing basketball. (Girl, rural area)	
Physical activity is active movements of our bodies. (Mother, suburban area)	
I think walking at the mall can be considered a physical activity as well (Father, urban)	
Physical activity that makes our bodies move more than the ordinary way and (we breath) more than normal breathing (Mother, urban area)	
Screen time, as far as I know, is 2 hours at most, isn’t it?. (Mother, rural area)	
(Question) How long should an adolescent sit in front of a TV or gadget? (Answer) Until I get tired of it (Boy, peri-urban)	
**Knowledge of overweight and obesity**	
(Overweight is) when the weight is not ideal compared to the height (Mother, urban area)	
It causes diabetes, heart disease, stroke, and back pain (Girl, urban area)	
(Question) Anything else that might happen to adolescents due to obesity? (Answer) Decreased self-confidence (Father, urban area)	
(Question) How can people achieve a healthier body weight? (Answer) Limit the screen time, exercise a lot, and eat less (Boy, peri-urban area)	
My weight once fell from 116 (kg) to 100 (kg), but it went up again to 105 (kg). (Question) What were you doing at that time? (Answer) I went to the fitness center regularly but it only lasted for 2 weeks (Boy, peri-urban area)	
At that time, it was only for a few months that I limited myself to not eating from morning till noon. At noon, I ate vegetables, stew, without rice. It only lasted for 2–3 months. I couldn’t handle it. I ate rice again.” (Boy, peri-urban area)	

The concept of a healthy menu consisting of ‘healthy four perfect five’ is an Indonesian concept of healthy eating that, first adopted in 1952, refers to four healthy food types [A staple food, a side dish, vegetables and fruits] with milk as the fifth. This adage was more prominently noted among participants than ideas around the importance of a balanced diet, especially in the rural and peri-urban populations.

*“*Healthy diet is ‘healthy four perfect five’*”* (Mother, peri-urban)

Most participants also understood the importance of fruit and vegetables and the hazard of too much fried food, although they could not always explain why fruits and vegetables are important or why too much fried food might be unhealthy.

*“*If you eat vegetables, sometimes it contains water, so it is good for our body*”* (Father, rural area)

*“*Fried food is unhealthy because sometime the oil has been used many times*”* (Girl, peri-urban)

Most participants were able to identify various sources of sugar and fat.

*“*Candy, chocolate, and coffee contain too much sugar*”* (Boy, urban area)

“(Source of fat is) milk. But fatty meal is meat. Chicken should not be eaten with the skin, only the breast is healthy*”* (Mother, peri-urban)

However, they had little understanding of recommendations around how much of these foods should (or shouldn’t) be eaten.

*“*I’ve heard about the balanced diet, but (I) don’t understand about the portion of each food group*”* (Father, peri-urban area)

*“*I can eat two cupcakes a day, but I can only have one tablespoon of sugar a day*”* (Girl, urban area)

Most participants were aware that physical activity included various activities of daily living in addition to sports or more intentional forms of physical activity.

*“*Activities that are done at home such as sweeping, washing, cooking … and also taking a walk as well as exercising*”* (Mother, rural area)

However, there was less certainty about the recommended amount of physical activity. The tentativeness reflected in many remarks (indicated by much use of expressions such as “maybe” or “isn’t it?”) suggested that many responses were largely guesses.

*“*Exercise for at least 60 minutes, isn’t it?” (Mother 14, peri-urban area)

“Maybe … it’s two or three times a week*?”* (Boy 13, peri-urban areas)

There was little appreciation of any recommendations around screen time, with most participants unable to answer questions about this at all.

*“*I honestly don’t know how long the maximum screen time is” (Father, peri-urban area)

Most study participants had some knowledge of the health effects of overweight and obesity. However, there were numerous misconceptions about this, including the best approaches to preventing obesity and weight management. For example, one peri-urban adolescent had tried a very strict diet to lose weight without any appreciation that weight management is a long-term goal that requires the development of a healthy lifestyle, rather than a short-term ‘quick-fix’ of weight loss.

*“*I limited myself to not eating from morning till noon. At noon, I ate vegetables, stew, without rice. It only lasted for 2-3 months. I couldn’t handle it” (Boy, peri-urban area)

For all areas of knowledge, parent and adolescent pairs appeared to have a similar level of understanding of the issues raised.

### Theme 2. Not a prominent daily concern

There was little evidence from parents or adolescents across the three sites that their knowledge influenced day-to-day decisions around shopping, meal choices, and daily activities (see Table 3). The responses from both adolescents and their parents showed that convenience and preference led their daily lifestyle choices. There was little evidence of planning for daily activities and a lack of motivation to practice according to their knowledge of healthy lifestyle.

“Breakfast at home, usually just something practical (such as) bread and milk” (Mother, urban area)

(Question) What is the hard part (about being physically active)? “I feel so lazy [laughs]” (Boy, rural area).

(Question) “What might help you to eat healthier food containing fruit and vegetables? Was any effort made at home?” “Nothing [laughs]” (Boy, rural area).

**Table 3:** Representative quotes of theme 2 (healthy lifestyle is not a daily concern)

(Question) What might help you to eat healthier food containing fruit and vegetables? Was any effort made at home? (Answer) Nothing [laughs] (Boy, rural)	
(Question) Why don’t you do badminton anymore? (Answer) I am bored (Question) Why is that? (Answer) Well, it’s just me being bored (Boy, rural)	
The thing is, now it is hard in terms of time. If I continue to do some physical activity, I cannot work as a motorbike taxi driver (Father, rural)	
It is difficult to find the time (for sports) (Boy, peri-urban area)	
I play with my cellphone all day whenever I have free time (Question) Apart from playing on your cellphone, are there any activities that you do? (Answer) No. That’s it (Boy, peri-urban area)	
(Question) Is it because you don’t think about it, or do you have a hard time eating fruit regularly? (Answer) I don’t think about it (Father, peri-urban)	
I want to (have a healthy lifestyle), but I was just like ‘ah, maybe later’ (Mother, peri-urban) The difficulty is that the children do not like doing physical activity. They are just not interested (Mother, urban)	
(Question) So, mostly it is fried dishes by the sound of it. Why? (Answer) Well, it’s the easiest (Girl, urban area)	
(Question) What are the challenges of preparing vegetables every day? (Answer) Well, I often forgot to buy. I have to frequently check the storage. The hard thing is (we) do not really like vegetables and fruits (Mother, urban)	
The challenge is that I am always tempted to order food through online services (Girl, urban)	

### Theme 3. Availability and accessibility

Issues around the availability and accessibility of healthy meals were significantly revealed in the interviews (see Table 4). Ultra-processed high-energy foods, such as chicken nuggets, were the food that urban and peri-urban parents reported always needing to have at home due to their practicality and easy accessibility.

“We always have frozen food such as nuggets and sausages because it is easy to prepare” (Mother, urban area)

**Table 4:** Representative quotes of theme 3 (availability and accessibility)

**Adolescent**	
Vegetables are rare. I eat kale if it is available. I eat cassava leaves if they are available at home.” (Girl, rural area)	
(Question) How can your parents help you to eat more vegetables? (Answer) Well it needs to be provided on the table (Boy, urban area)	
If I am hungry and do not know what to eat, I drink packaged apple juice which is always available (Girl, urban)	
I usually drink packaged sugary drinks. It is always available at home. I rarely eat something sweet like candy since it is not available. (Boy, urban area)	
**Parents**	
It (fruit) is a bit difficult. (Question) Why is it difficult, Sir? (Answer) We have to plan them. It is too far to buy them. It cost a lot. (Father, rural area)	
In the market, the choices are either banana or papaya. Sometimes we can get bored too. (Mother, rural)	
(Question) Why do you rarely play badminton? (Answer) Well now, there is no accessible field nearby anymore (Mother, peri-urban)	
The difficulty is the lack of space. Moreover, there are too many cars around here. Thus, it is difficult (to do physical activity).” (Mother, urban)	
Fruit is only available in the early days [after the monthly salary payment] (Mother, urban)	

Most adolescents said that they did not eat healthy meals because they simply ate what was provided for them at home.

“Well, just whatever is available. If there is fish, I eat fish. If there is egg, I eat egg” (Boy, rural area)

In contrast, while at some level, many parents expressed a desire to prepare healthier meals, because healthier foods were not always eaten by their children, many suggested that this lead over time to them becoming dissuaded from preparing healthy food.

“When it is not fried, no one eats it, so it becomes wasteful” (Mother, peri-urban area).

For parents, accessibility was also influenced by the affordability of food, both in terms of its price and location (time, travel costs), especially for those in the rural area.

(Question) “Do you have vegetables every day *“*No, it is hard. Only if there is someone who sells it around here.*” (*Question) “Why don’t you buy them at the market, Sir?” *“*It’s costly. The market is far from here, near the port*”* (Father, rural area)

Lack of availability of an enabling environment for a more active lifestyle was also commonly cited by both adolescents and parents, across all participating sites, as a barrier to greater participation in physical activity.

*“*The environment also plays a role. For example, it is dangerous to ride a bike because there are a lot of motorbikes*”* (Mother, urban area)

*“*The badminton field is gone now. It was changed into a building. We have no place to play badminton again*”* (Father, rural)

### Theme 4. Limitations in parenting skills

From each region, participant responses showed limitations in parents’ understanding of adolescent development, especially around the development of autonomy and independence (see Table 5). Parents were permissive to a very high level around their children’s food choices and daily activities. There was no evidence that parents tried to regulate their children’s behaviors through interactive negotiation. Instead, parents swung from a highly permissive stance to one of prohibition when parents became more concerned about their adolescent’s behaviors.

*“*There are none (rules). He can eat whatever he wants*”* (Father, rural area).

*“*I do not forbid it, yet when it is too much, for example drinking boxed tea or sweet things continuously, I will stop them*”* (Mother, urban area).

**Table 5:** Representative quotes of theme 4 (limitation in parenting skills)

**Lack of interactive discussion on healthy lifestyle**	
(Question) What do you usually discuss with your son about food? (Answer) Sometimes I ask him to eat fresh fruit but otherwise, I suggest he eats whatever is available (Father, rural area)	
We rarely discuss things (Father, peri-urban area)	
(Question) Do you ever discuss food or daily activity at home with your parents? (Answer) No. My mother only asks me what I want to eat tomorrow (Boy, urban area)	
**Lack of structure and rules / expectation**	
(Question) Is there any regulation on eating and drinking? For example, you cannot eat or drink certain things.” (Answer) There is no regulation for food. (Mother, rural area)	
(Question) What are the rules (on screen time)? (Answer) Turn it off when it is midnight (Boy, rural)	
(Question) What are the regulations? (Answer) Well, do not play with your cellphone too much for it can disturb your study (Question) How much is too much for you, Mam? (Answer) If I see him on his gadget continuously (Mother, rural)	
Sometimes, if it is too much, the (TV) remote control is taken. (Laughs) Then I turn it off (Father, peri-urban)	
(Question) Is there any method to restrict unhealthy food or drinks, Sir? (Answer) There is no specific method, at least we do not store any (unhealthy food). (Father, urban)	
**Lack of parental modeling**	
The father is still sleeping when the children ask for a morning run or bike (laughs). (Mother, peri-urban)	
My parents do not eat broccoli either. Thus, why I should like it? (Boy, urban)	
His father loves snacking so much, so they (father and son) both are fit each other, (Mother, urban)	
Daddy keeps on playing with his phone. Mom does it (physical exercise) sometimes (Girl, urban area)	
**Gender inequity in parenting**	
(Question) Who cleans the house? (Answer) Mother (Question) Who does the dishes? (Answer) Mother (Question) What about your father? (Answer) He works. (Boy, rural area)	
The mother does all the house chores (Father, rural)	
If I remind him, he does not listen. Yet, if the mother says it to him, he listens. Thus, his mom talks to him more often (Father, rural)	
The eating habit is always monitored by the mother (Father, peri-urban)	
I do not know about balanced diet. Maybe my wife (know), she is the one who usually learns about food and health from youtube. (Father, peri-urban)	

Furthermore, several statements from parents indicated that their daily practices around eating and physical activity were primarily driven by their children’s preferences.

*“*We mostly follow (what the children like to eat)” (Father, peri-urban area).

Surprisingly (as this was not the focus of the interviews), the extent of gender imbalance around parenting roles featured prominently in the interviews with parents and adolescents from the rural and peri-urban regions. Our study questions did not specifically address gender roles around meals and physical activities or the relative balance of parenting responsibilities between parents. While this may also have been a feature in urban families, it did not arise in any of those interviews. Mothers played a dominant role in meal planning, ensuring that food was available at home including shopping and cooking, as well as monitoring their children’s eating and physical activity when it was monitored. There was almost no adolescent involvement in household chores, including grocery shopping and meal preparation.

*“*I only have sons. Thus, I do all the household chores. No one helps me at home*”* (Mother, rural area).

“Well, the rules (around eating) are given and monitored by their mother*”* (Father, peri-urban).

## Discussion

Parents and adolescents across three different sociodemographic backgrounds had basic knowledge about healthy lifestyles, albeit with some major knowledge gaps, especially around the benefits of physical activity and the downsides of sedentary activity. Consistent with studies from other countries in Asia [14, 15], the most common understanding of a healthy diet related to the importance of eating a range of foods rather than an appreciation of the value of different types of food or knowledge of recommended amounts. In Indonesia, the government’s 1952 health promotion message about nutrition of ‘healthy four perfect five’ remains frequently used by families to justify the importance of eating a variety of foods [16]. One concern is whether such general information about a balanced diet is sufficiently granular or practical to help contemporary families make healthy choices in the context of current food availability and the rapidly rising prevalence of overweight and obesity. Rather than knowledge, consistent with a recent global qualitative review [12], family dietary practices were highly influenced by preference, convenience, availability and price.

There was little confidence about any recommendations or expectations of daily physical activity, with a high level of tentativeness of parent responses. These findings are similar to other qualitative studies, such as from Morocco which found that while parents and adolescents generally understood the benefits of exercise, they had little appreciation of the ideal frequency and duration of physical activity for adolescents [17]. While parents were also aware that their children had high levels of screen time, consistent with other recent studies from India and Malaysia [14, 18], there was little evidence that parents tried to encourage or shape more active daily routines at home.

In this study, parents and adolescents largely understood the causes of overweight and obesity, with some understanding that adolescents needed to change some of their behaviors. However, there was little confidence that this might occur. For example, these overweight adolescents wanted strategies to help them to immediately lose large amounts of weight, just like a study in East Java in which 78% of 206 female adolescents with an unhealthy diet reported wanting to acutely lose weight [19]. While this can be appreciated to reflect adolescents’ short-term reward-seeking behaviors [20], it also demonstrates a striking lack of appreciation that the core aim of obesity management is shaping long-term healthy lifestyles [9]. Our findings provide little confidence that parents appreciate the importance of their role in helping to influence their children’s behaviours around this. It also reinforces the importance of adolescents’ agency around food choices and how parents engage with their adolescents around this [21].

Availability and accessibility are strong determinants of eating behaviours and physical activity that reflect the influence of socio-economic conditions [22, 23]. Study participants from the rural area were found to be most affected by these factors, especially around limited accessibility to healthy food, as previously found [14, 24]. However, beyond these factors, *“*not having time” and “lazy” were also the common explanations used by parents for not preparing more healthy food and for their children having highly sedentary lifestyles. Aspects of convenience such as easy access, low price and practicality also drove parent choices, as did adolescent preferences, consistent with other studies about food choices [25, 26]. This finding emphasizes that developing enabling environments, including access to affordable healthy food, is an important aspect of establishing healthy diets [23, 27].

There are two elements to parenting. One is about the aspect of demand and structure, while the other is about responsiveness and support [28]. The findings from this study suggest poor parental ability to provide both structure and support for healthy lifestyles due to the absence of clear expectations and rules, the lack of parental ability to make healthy food available, the lack of parental modeling, and the lack of communication skills that would enable them to negotiate with their children around aspects of healthy lifestyles. In the same way, the finding that adolescent-parent dyads mostly had similar levels of knowledge, rather than higher levels, reduces the ability of parents to be a source of information for their children. These challanges suggest lack of information, skills and support for parenting in Indonesia, especially support for parenting contemporary adolescents, who face a very different set of challenges than they faced when young [29]. These findings also reveal a lack of environmental enabling factors that might otherwise empower parents to develop a healthy lifestyle at home, supported by government policies or programs that influence social norms and the resources available for healthy lifestyles, as found in a study from China [30].

While not the primary focus of this study, we found substantial gender inequalities between fathers and mothers around parenting within socioeconomically disadvantaged families. Consistent with other studies, [31] we found that fathers were largely absent from both practical and emotional aspects of caregiving. Although a review showed that father and child physical activity are modestly associated [32], another recent systematic review revealed that a father’s eating habits, weight status, and parenting techniques influence dietary behaviors of their children [33].

This study has a number of limitations. We were interested in understanding the modifiable behaviours of overweight and obesity within the home environment with the objective of informing a future intervention in primary care. As such, our inclusion criteria was for adolescents with overweight or obesity. We do not know to what extent the knowledge, attitudes and skills highlighted in these dyads are reflective of the attitudes of adolescents of more normal weight and their parents. The consistency of our findings with previous studies suggests they may be similar. We also did not explore the extent to which these adolescents had engaged with primary care providers around their weight to know to what extent their knowledge, attitudes and skills may have reflected engagement with the health care system. Consistent with the majority of studies of adolescents and their health, more mothers than fathers participated in these interviews as mother were more commonly present at the time of data collection. Nevertheless, we included father(s) from each sociodemographic setting. These families were recruited from three different sociodemographic backgrounds. While the small sample size and diversity of the Indonesian population means that these results are not able to be used to describe lifestyle and parenting challenges around raising adolescents across Indonesia, an unexpected finding was that their lifestyle practices were not particularly different by setting.

## Conclusion and future implication

This study found deficiencies in the knowledge, attitudes and capabilities of Indonesian parents of adolescents with overweight or obesity to implement healthy lifestyles, and also identified that the agency that adolescents have around lifestyle choices then influences family decisions. Barriers around the availability and accessibility of healthy food and physical activity facilities featured more in rurally based families than the urban families. Future obesity prevention program should consider providing more practical information about healthy lifestyles, and include interventions to build motivation in parents and adolescents. Efforts to provide families and communities with the nutritional, social and physical environments that support sustained behavioral change in adolescents also appear indicated. This will equally require efforts to improve parenting capabilities as parents remain the primary actors shaping enabling environments for adolescents.

## Data Availability

Data and materials used for this study are available from the corresponding author upon request.

## Abbreviations

BMI: Body Mass Index
WHO: World Health Organization
